# Genomic analysis reveals depression due to both individual and maternal inbreeding in a free‐living mammal population

**DOI:** 10.1111/mec.13681

**Published:** 2016-06-06

**Authors:** Camillo Bérénos, Philip A. Ellis, Jill G. Pilkington, Josephine M. Pemberton

**Affiliations:** ^1^ Institute of Evolutionary BiologyAshworth LaboratoriesKing's Buildings, Charlotte Auerbach RoadEdinburghEH9 3FLUK

**Keywords:** genomic data, heterozygosity fitness correlation, inbreeding, inbreeding depression, maternal inbreeding depression

## Abstract

There is ample evidence for inbreeding depression manifested as a reduction in fitness or fitness‐related traits in the focal individual. In many organisms, fitness is not only affected by genes carried by the individual, but also by genes carried by their parents, for example if receiving parental care. While maternal effects have been described in many systems, the extent to which inbreeding affects fitness directly through the focal individual, or indirectly through the inbreeding coefficients of its parents, has rarely been examined jointly. The Soay sheep study population is an excellent system in which to test for both effects, as lambs receive extended maternal care. Here, we tested for both maternal and individual inbreeding depression in three fitness‐related traits (birthweight and weight and hindleg length at 4 months of age) and three fitness components (first‐year survival, adult annual survival and annual breeding success), using either pedigree‐derived inbreeding or genomic estimators calculated using ~37 000 SNP markers. We found evidence for inbreeding depression in 4‐month hindleg and weight, first‐year survival in males, and annual survival and breeding success in adults. Maternal inbreeding was found to depress both birthweight and 4‐month weight. We detected more instances of significant inbreeding depression using genomic estimators than the pedigree, which is partly explained through the increased sample sizes available. In conclusion, our results highlight that cross‐generational inbreeding effects warrant further exploration in species with parental care and that modern genomic tools can be used successfully instead of, or alongside, pedigrees in natural populations.

## Introduction

Inbreeding depression, the reduction in fitness or fitness‐related trait values as a result of mating between related individuals, has intrigued evolutionary biologists for many decades. The genetic basis is not clearly understood, but is generally believed to be due to two phenomena which are driven by consanguinity: expression of (largely) recessive deleterious alleles or increased homozygosity at overdominant alleles. The relative importance of each is still a topic of debate, as few experiments are able to tease apart the two effects (Charlesworth & Willis 2009).

While there is ample evidence for inbreeding depression, as it has been documented in a wide range of plant and animal taxa (Keller 1998; Szulkin *et al*. 2007; Grueber *et al*. 2010; Laws *et al*. 2010; Walling *et al*. 2011; Benesh *et al*. 2014), much of the evidence is generated in controlled environments using experimental crosses or comes from self‐fertilizing plants (Weller *et al*. 2005). Much less evidence for inbreeding depression exists in wild populations with naturally occurring inbreeding, especially in vertebrates, and several ideas have been put forward to explain this relative deficiency (Keller & Waller 2002). First, inbreeding might be relatively rare in natural populations, and second, sample sizes are too small to contain a sufficient number of highly inbred individuals to detect inbreeding depression (Csilléry *et al*. 2006). Additionally, the calculation of inbreeding coefficients (*F*) requires pedigrees, which are laborious to obtain and even with genetic support, are often incomplete and contain errors, the net result of which is an underestimate of individual inbreeding coefficients (Pemberton 2004, 2008).

Being inbred might not only have detrimental effects on an individual's own fitness, but also on its offspring. Such indirect effects of inbreeding status could, for example, arise in systems where maternal effects are important contributors to offspring phenotype. Evidence for maternal effects in natural populations is plentiful (Reznick *et al*. 1996; Wilson *et al*. 2005a; Beckerman *et al*. 2006; Rasanen & Kruuk 2007), and while an increasing number of papers are documenting, a additive genetic basis to these maternal effects (McAdam *et al*. 2002; Wilson *et al*. 2005a), to our knowledge there have been few investigations of, and there is little evidence for, maternal inbreeding effects on offspring traits in unmanaged natural populations (Laws *et al*. 2010). However, a parallel study to the present one on red deer has documented strong effects of both offspring and maternal genomic inbreeding on survival to recruitment (Huisman *et al*. 2016).

A possible explanation is that due to selection on inbred individuals, individuals that survive to reproduce are fewer and less inbred than juveniles, making it harder to detect inbreeding depression among mothers. Also, in natural populations, pedigree information is generally poorer for mothers than for their offspring, causing greater underestimation of their inbreeding coefficients and thus compromising the ability to detect inbreeding depression.

To circumvent the need for pedigrees in natural populations, genotypes at molecular markers have been used as an alternative to pedigree construction for investigating inbreeding depression. For example, individual multilocus heterozygosity, often at a small sample of microsatellite loci (e.g. 10–20), has been correlated with fitness or fitness‐related traits (Coulson *et al*. 1998; David 1998; Amos *et al*. 2001; Chapman *et al*. 2009). This approach (known as heterozygosity–fitness correlation, or HFC) relies on the assumption that heterozygosity at marker loci is correlated with inbreeding and thus homozygosity at functional loci harbouring deleterious recessive alleles. A meta‐analysis of 628 reported effect sizes found that overall, there is a weak effect of heterozygosity on trait measures, but that the literature contains biased reporting, with effect size dwindling with sample size (Chapman *et al*. 2009).

All else being equal, the precision of genomic estimates of inbreeding will scale positively with the number of markers used (Slate *et al*. 2004). Until very recently, most studies of natural populations used a modest number of microsatellite markers. While HFCs are routinely reported even when very few markers are used (Chapman *et al*. 2009), generally heterozygosity is only weakly correlated with pedigree inbreeding coefficient (Slate *et al*. 2004) or with heterozygosity at other markers (heterozygosity–heterozygosity correlations between subsamples of markers), indicating that in general such studies do not capture inbreeding very well (Balloux *et al*. 2004).

Recent genomic advances have made it possible to screen individuals for tens of thousands of single nucleotide polymorphisms (SNP) relatively affordably. It has recently been shown using 13 198 SNP markers that, in a captive population of oldfield mice (*Peromyscus polionotus*), heterozygosity was correlated with pedigree inbreeding coefficient and showed high identity disequilibrium, that is there was high covariance in heterozygosity among markers within individuals (Hoffman *et al*. 2014). In the same paper, marker heterozygosity at 14 585 SNP loci was correlated with fitness (dying before age 1 year vs. dying at an older age) and a fitness‐related trait (parasite load) in a natural population of harbour seals (*Phoca vitulina*), suggesting that heterozygosity at thousands of markers can be used to detect inbreeding depression in the absence of a pedigree.

Theoretically, genomic estimates obtained using thousands of markers could have several advantages over pedigrees, as the pedigree‐derived inbreeding coefficient is merely the expectation of the proportion of the genome which is identical‐by‐descent (IBD). Even with an extensive and complete pedigree, the realized proportion of the genome which is IBD will differ from this expectation due to Mendelian sampling and recombination. With genomic information, realized inbreeding coefficients can be estimated, enabling greater individual‐level precision than the pedigree (Powell *et al*. 2010; Yang *et al*. 2010; Hill & Weir 2011; Kardos *et al*. 2015). The revolution in high‐throughput SNP genotyping in humans and farm animals has led to rapid expansion of interest in detection of inbreeding effects from these data, with many studies now reporting inbreeding effects, particularly in humans, based on large samples of unrelated subjects originally genotyped for genomewide association studies (McQuillan *et al*. 2012; Verweij *et al*. 2014; Joshi *et al*. 2015).

However, there are few papers which have examined the relative performance of the different genomic estimators now available, and it is unclear which is the most powerful and preferred estimator to study inbreeding depression in natural populations. Multilocus heterozygosity (MLH; or its inverse, homozygosity) has been used since the allozyme era and is easy to calculate, as it does not require knowledge of population‐wide allele frequencies. However, the scale of this estimate is very different from pedigree inbreeding, and its relationship with pedigree‐derived inbreeding coefficients may be both population‐ and marker‐specific. In consequence, many estimators have been derived which account for allele frequencies [e.g. based on the excess homozygosity (Purcell *et al*. 2007)] and produce an estimator on a scale much more comparable to pedigree inbreeding. A recent estimator which was developed for bi‐allelic markers gives more weight to rare homozygotes by scaling by the expected heterozygosity at each marker locus. *F*_hat3_ in (Yang *et al*. 2011) is claimed to be the most accurate SNP‐by‐SNP‐based estimator to date (Keller *et al*. 2011; Yang *et al*. 2011). As this measure estimates a correlation coefficient between unifying gametes rather than the proportion of the genome IBD, the range extends beyond the 0–1 range we would expect for an IBD‐based estimator and negative values are observed. An alternative and popular method of estimating individual inbreeding, especially in the field of human genetics, is based on runs of homozygosity (ROH) (Broman & Weber 1999; Gibson *et al*. 2006; McQuillan *et al*. 2008). This method detects and quantifies consecutive stretches of the genome which are homozygous above a user‐defined length, and as these are likely to be autozygous, it is supposed to capture autozygosity at causal loci (which are typically believed to have lower minor allele frequencies than genotyped SNPs) found within these runs with more precision than either pedigree or SNP‐by‐SNP‐based estimators (Keller *et al*. 2011). In addition, in contrast to other genomic estimators of *F*, its scale is directly comparable to pedigree *F* as it estimates the proportion of the genome IBD.

Simulations using a range of parameters representative of a human demographic scenario have shown that ROH outperforms all other (genomic and pedigree) methods when it comes to detecting inbreeding load due to rare deleterious recessives (Keller *et al*. 2011). A more recent paper explored how well various estimators of inbreeding correlated with the ‘true’ proportion of the genome IBD in a scenario more typical of populations with smaller population sizes (Kardos *et al*. 2015). It was shown that while all genomic estimators were superior to the pedigree, ROH‐based estimators were not necessarily more precise than SNP‐by‐SNP‐based estimators of inbreeding (Kardos *et al*. 2015). Further studies examining multiple genomic estimators of inbreeding have shown that both ROH and homozygosity may independently affect human height (McQuillan *et al*. 2012), and that several genomic estimators of inbreeding (homozygosity, *F*_hat3_ and a ROH‐based estimator) all had similar power to detect inbreeding depression in both fitness and production traits in livestock populations, but always outperformed pedigree inbreeding coefficients alone (Bjelland *et al*. 2013; Pryce *et al*. 2014). Hence, there appears to be little consensus as to which genomic estimator to use when estimating inbreeding depression.

The Soay sheep (*Ovis aries*) is a primitive breed of domestic sheep, and an unmanaged population living on the Scottish island of Hirta, St Kilda, has been studied intensively since 1985 (Clutton‐Brock & Pemberton 2004). The whole‐island population size is relatively small, fluctuating between ~600 and ~2100. A recent estimate of effective population size, derived from SNP data, is 194 (Kijas *et al*. 2012). As is typical for ungulates, there is substantial reproductive skew in males, as dominant males monopolize access to females (Preston *et al*. 2003). As this creates large paternal (half) sibships, as most females and some males are philopatric, and since generations overlap, there is a potential for mating between close relatives and thus inbreeding depression. As adult females live for several years and breed virtually every year, there are also large maternal sibships (Clutton‐Brock & Pemberton 2004), which enables the effects of an individual's own inbreeding coefficients to be teased apart from those of its mother's inbreeding.

Two previous studies have explored whether inbreeding depression is present in the St Kilda Soay sheep. An early study showed an HFC between microsatellite MLH and a measure of parasite resistance (August strongyle faecal egg count) and overwinter survival, consistent with the observation that heterozygosity increased with age (Coltman *et al*. 1999). A more recent study investigated whether maternal or individual inbreeding depression could be detected in neonatal traits using pedigree *F* and microsatellite MLH, but failed to detect any inbreeding depression in either birthweight or neonatal survival, which was explained by a low mean and variance in inbreeding and a weak correlation between MLH and pedigree *F* (Overall *et al*. 2005). While of great interest at the time, by today's standards these studies can be seen as underpowered in a number of respects, especially in terms of marker number. Since the previous studies were published, progress has been made in several key aspects of data collection, warranting revisiting the analysis of inbreeding depression in Soay sheep. The phenotypic and life‐history data set has grown substantially, and extensive improvements to the accuracy and completeness of the pedigree have been made, resulting in more accurate fitness measures and inbreeding coefficients, and thousands of sheep have now been genotyped at 37 047 autosomal SNP markers, giving a much better coverage of the genome than the panels of microsatellites used previously. These changes enable us to finally examine whether and how maternal and/or individual inbreeding affect fitness components and fitness‐related traits, and compare estimators of inbreeding in the detection of inbreeding depression.

The objectives of this study were to (i) investigate the effects of maternal and offspring inbreeding on juvenile body size and fitness, (ii) examine whether multilocus heterozygosity and other genomic estimators of individual inbreeding are as or more able to detect inbreeding depression than pedigree‐derived inbreeding coefficients and (iii) explore which of the genomic estimators of inbreeding show the strongest association with trait values. We estimated inbreeding depression in three early‐life morphological traits which are correlated with fitness in Soay sheep (Milner *et al*. 1999; Jones *et al*. 2005; Wilson *et al*. 2005b), one of which is measured in neonates (birthweight) and two of which are measured in 4‐month‐old lambs (measures taken during August: hindleg and weight). We also examined the effects of inbreeding on first‐year survival and two annualized fitness components for sheep surviving past age 1 (annual survival and annual breeding success). In most of our analyses, we compared pedigree and genomic metrics from a practical point of view as they would arise in a typical study of a free‐living population with an imperfect pedigree. Specifically, we were able to estimate genomic inbreeding coefficients for individuals with insufficient pedigree information to be retained in the pedigree‐based analyses, resulting in larger sample sizes in the former group. Any differences between pedigree‐ and genomic‐based inbreeding effects are thus potentially the result of a combination of both increased sample size and improved estimation of inbreeding coefficients. We reran some analyses using identical sample sets for pedigree and genomic inbreeding to shed light on these possibilities.

## Methods

### Study system and morphological data collection

The Soay sheep breed is descended from the first sheep brought to the British Isles during the Bronze Age, but it has also experienced an admixture event with the Dunface sheep breed in the 19th century (Feulner *et al*. 2013). Sheep resident in the Village Bay area of Hirta, St Kilda (NW Scotland), where approximately one‐third of the sheep inhabiting the island of Hirta are found, have been the subject of a long‐term individual based study since 1985 (Clutton‐Brock & Pemberton 2004). Most individuals (ca. 95%) are captured, ear‐tagged and weighed within a few days of birth. Every August, ~60% of resident sheep are captured and several morphometric measures are taken, including hindleg and body weight. Winter mortality is monitored, with the peak of mortality occurring at the end of winter/early spring, and ca. 80% of all deceased sheep are found.

### Collection of fitness data and pedigree construction

Parentage was inferred through a combination of observational data and molecular markers for maternal links, and using molecular markers only for paternal links (Johnston *et al*. 2013; Bérénos *et al*. 2014). Molecular parentage assignments were predominantly (for 4371 individuals) obtained using 315 polymorphic and unlinked SNP markers [assigned with 100% confidence in the r package *MasterBayes* (Hadfield *et al*. 2006)], but where SNP genotypes were not available for either lamb or candidate fathers, paternity was assigned using 14–18 polymorphic microsatellite markers [for a total of 222 lambs, assignment with confidence greater than 95% in *MasterBayes* (Morrissey *et al*. 2012)]. This enabled the construction of a pedigree with a maximum depth of 10 generations and consisting of 6740 individuals, of which 6336 were nonfounders. More pedigree summary statistics and details about pedigree construction can be found in Bérénos *et al*. (2014) and Johnston *et al*. (2013).

The selection of individuals to include in a study of inbreeding depression using pedigree inbreeding coefficient, *F*_ped_ is complex in the presence of imperfect pedigree information and especially, uneven amounts of pedigree information between individuals. If very stringent minimal criteria (in terms of the number of generations and links of pedigree information) are set then *F*_ped_ estimates will be relatively accurate but sample sizes may be small. If very relaxed minimal criteria are set then the sample will contain some individuals with inaccurate values of *F*_ped_ (specifically: some inbred individuals will have *F*_ped_ = 0) but sample sizes will be larger. In addition, a species' biology may affect which pedigree links are more likely to be known; in Soay sheep, matrilineal links are more likely to be observed and genetically confirmed as females do not routinely disperse from the study area. We conducted an analysis of the implications of different minimal pedigree information criteria (Tables S1 and S2) and concluded that for the main analyses described below, a relaxed minimal criterion, namely both parents and at least one maternal grandparent known, resulted in the best compromise between accuracy and sample size. The effect of varying this criterion is stated where relevant.

### Genotype data

Individuals were genotyped using the Ovine SNP50 BeadChip (Illumina) using an iScan instrument at the Wellcome Trust Clinical Research Facility Genetics Core (Edinburgh, UK). Quality control was performed in plink (Purcell *et al*. 2007). Individuals with call rate >95% were retained, and loci with minor allele frequency <0.01, call rate <99%, or which deviated from Hardy–Weinberg equilibrium (HWE) at *P* < 1e‐05 were all discarded. A total of 5805 individuals and 37 037 autosomal SNP loci passed quality control. Median spacing between SNPS was 50.2 Kb, and neighbouring SNPs were generally in high linkage disequilibrium (LD, mean *r* ^2^ = 0.3).

### Inbreeding estimators

For each individual, we calculated four different estimates of inbreeding.

1. *F*_ped_: Expected *F* from the pedigree, calculated in the r package *Pedigree*.
2. *F*_hom_: Proportion of successfully genotyped loci which were homozygous.
3. *F*_GRM_: A genomewide estimate of inbreeding, which is a weighted average across all loci [Fhat3 in (Yang *et al*. 2011)] and was calculated in the gcta software (Yang *et al*. 2011). This estimator gives more weight to homozygotes of the minor allele than to homozygotes of the major allele at each locus and has a lower sampling variance than other homozygosity‐based single SNP measures (Yang *et al*. 2011). *F*_GRM_ for each SNP *i* and individual *j* is calculated as follows:

$$F_{\mathrm{GRM}}=\frac{x_{ij}^{2}-\left(1+2p_{i}\right)x_{ij}+2p_{i}^{2}}{2p_{i}\left(1-p_{i}\right)}$$

where *x* is the number of copies of the reference allele (0, 1 or 2) and *p* is the population‐wide allele frequency of the reference allele.

*F*_ROH_: The proportion of the genome (the total physical length of all autosomes from the first to the last SNP marker: 2 434 125 base pairs) which is found in runs of homozygosity (ROH). A run of homozygosity was here defined as a stretch of DNA of length greater than 5 Mb which was completely homozygous at the genotyped SNPs. The expected lengths of a ROH segment generated by a single path should follow an exponential distribution with mean 1/2 *g* M, where *g* is the number of generations from the common ancestor (Fisher 1954) and M is genome size in Morgans. Assuming that 1 cM ≈ 1 Mb, we expect that ROH of 5 Mb are the result of a common ancestor 10 generations ago. We have chosen a minimum length of 5 Mb following recommendations in Purfield *et al*. (2012) as it was shown that in dairy cattle, with an N_e_ comparable to Soay sheep, ROH of this length could accurately be detected at this marker density, but that marker density is too sparse to reliably detect shorter ROH reflecting more distant inbreeding events. As setting ROH thresholds is unavoidably arbitrary, we have, for a select few traits, also tested whether setting a minimum length of 10 Mb would affect inbreeding depression estimates.

Prior to the detection of ROH, SNPS with low‐frequency minor alleles (MAF < 0.05) were excluded and genotype data were pruned for LD in plink (Purcell *et al*. 2007) setting the variance inflation factor to 10, which approximately corresponded to *r* ^2^ = 0.9), resulting in a data set consisting of 13 370 SNPs with mean and median spacing between adjacent SNPs of 186Kb and 131Kb, respectively. LD pruning was carried out following the recommendations in (Howrigan *et al*. 2011; McQuillan *et al*. 2012) as simulations have shown that LD pruned ROH show the strongest correlation with autozygosity (Howrigan *et al*. 2011). When failing to prune for LD, homozygous stretches could occur by chance rather than autozygosity and are less likely to harbour rare (partially) recessive deleterious alleles in a homozygous state (Howrigan *et al*. 2011). ROHs were found using plink using the following commands: –homozyg‐window‐kb 200000 –homozyg‐window‐snp 5 –homozyg‐window‐het 0 –homozyg‐snp 15 –homozyg‐density 275 –homozyg‐kb 5000 –homozyg‐gap 1000 –homozyg‐window‐missing 2. This resulted in ROH with a median length of 36 SNPs (range: 19–386) and 6.6 Mb (range: 5–67 Mb).

### Estimation of inbreeding depression

For information on the measurement of birthweight, August hindleg and August weight, see (Beraldi *et al*. 2007). We analysed these continuous traits using linear mixed models (so‐called animal models) in asreml‐r (Gilmore *et al*. 2009) because previous research has demonstrated that in Soay sheep they harbour significant variation due to additive genetic variation, maternal additive genetic variation and maternal environment (Bérénos *et al*. 2014) and (more broadly) that if both additive genetic and inbreeding effects are present, estimating *V*_a_ and inbreeding depression simultaneously yields less biased estimates of each (Becker *et al*. 2016). Both individual *F* and maternal *F* were fitted as fixed effects. Consequences of varying the minimal amount of pedigree information criterion for the *F*_ped_ analyses were investigated and are described in the Supporting information.

A comprehensive list of fixed effects fitted, known from previous analyses of juvenile body size traits (Bérénos *et al*. 2014), is shown in Table S3 (Supporting information). The models included lambs of both sexes as sex differences in these traits are small and easily dealt with by a fixed effect of sex. Random terms included an additive genetic effect, a year of birth effect, a maternal additive genetic effect and a maternal environmental effect representing remaining effects due to the identity of the mother (Table S3, Supporting information), which have also all previously been shown to contribute significantly to trait variance (Bérénos *et al*. 2014). To standardize additive and maternal additive genetic effects across all analyses, fitted relatedness matrices were calculated using the pedigree. Statistical significance of fixed effects was assessed using Wald F statistics.

We next examined the effects of inbreeding on first‐year survival annual survival, AS, and annual breeding success, ABS. As males and females have very different survival rates (males survive less well than females) and reproductive scheduling (females produce 0–2 lambs every year while males produce 0–22 per year, with nonzero values mainly occurring in older males), we modelled the fitness components separately for each sex. First‐year survival was a binary response variable describing whether an individual survived past May 1st in the year following birth. For each sheep year *j,* AS was a binary response variable describing whether or not an individual survived past May 1st in year *j *+* *1, and ABS was defined as the number of offspring born in year *j*. We adopted this analytical approach, rather than studying lifespan or lifetime breeding success for several reasons. First, because in the study population, variation in population density and weather conspire to cause high variation in survival and fecundity between years (Coulson *et al*. 2001), such that lifetime measures of fitness are strongly associated with year of birth, that is they include a high element of luck (Coltman *et al*. 1999). In these circumstances, it is more straightforward to model annual survival and breeding success, fitting a fixed effect of age and a random effect of year, than to model lifetimes fitting covariates for age and year (or multiple random effects of year). Furthermore, it enabled us to include data for individuals for which we have information for some but not all years (e.g. fringe animals or animals that are missing but not confirmed dead: for each individual, AS and ABS were only estimated for those years an individual was observed and classified as being a part of the study population. Lastly, we were interested in how inbreeding affected the two key fitness components (survival: does an individual survival; breeding success: given that it survives, how many offspring does it produce) separately for lambs (that do experience maternal care) and adults, as doings so would allow us to test whether inbreeding affects the different life‐stages and fitness components differently. All fitness components were analysed using generalized linear mixed models (GLMM) with a Bayesian approach using Markov chain Monte Carlo (MCMC) algorithms in the r package *MCMCglmm* (Hadfield 2010). We analysed the fitness components in a Bayesian framework, because they have non‐normal distributions: a categorical (binomial) error distribution was used for first‐year survival and annual survival, and a Poisson's error distribution was used for annual breeding success. We did not use an animal model approach for the fitness components because previous research suggests negligible additive genetic variance for these traits as well lifetime breeding success (Morrissey *et al*. 2012; Johnston *et al*. 2013). For models analysing fitness components, in addition to the individual and maternal inbreeding coefficient, fixed effects for the focal individuals were chosen based on Bérénos *et al*. (2015) and Johnston *et al*. (2013) and subsequent exploratory analyses (for a full list of fixed effects fitted, please see Table S3, Supporting information). Birthweight was included in the models of first‐year survival as we were interested in the effects of inbreeding on fitness over, and above the effects, it may have through reduced birthweight (Jones *et al*. 2005). Birthweight was corrected by taking the residuals from a model containing a significant third‐order polynomial which best described the relationship between age at capture (in days, only including individuals that were captured 10 days postbirth or earlier) and weight at capture (in kilograms). Random effects included are shown in Table S3 (Supporting information). To accommodate for differences in model complexity and data structure between models, MCMC chain length varied between the models, but all chains were run for at least 6 000 000 iterations with a burn‐in phase of at least 2 000 000 iterations, and at least 2000 independent samples were taken from the posterior at equally spaced intervals. Priors were specified for random effects, such that the total phenotypic variance was divided equally between the random effects fitted, and for survival, residual variance was fixed at one. Exploratory analyses suggested that model estimates are not dependent on the priors used. Convergence was assessed by visual inspection of the traces and was deemed acceptable if autocorrelation between successive samples was below 0.05. Results are presented as posterior modes of the sampled iterations and the 95% credibility interval. Significance of effect sizes can be assumed if the 95% credibility interval does not overlap with zero.

## Results

### Variation within and between different inbreeding estimators

Using all 6336 nonfounding individuals in the pedigree, 12.8% of individuals (*n* = 813) had nonzero *F*_ped_. As expected, this is an underestimate of the prevalence of inbreeding, as the proportion of individuals with nonzero *F* increased to 21.3% when only individuals with both parents and a maternal grandmother were known and to 41.2% when only individuals with four known grandparents were considered (Table S1, Supporting information). Imposing more stringent ancestry criteria on the data comes at a cost, as the total number of inbred individuals decreases with each additional restriction. As a result of these data set restrictions, the standard deviation in *F*_ped_ increased from 0.018 to 0.022 to 0.028. While many nonzero *F* values were very small indicating that we were sometimes able to detect the occurrence of mating between distantly related individuals (the smallest *F* value was 6.1 × 10^−5^), there was also evidence for close inbreeding. 112 and 54 sheep had *F* values equal to or greater than 0.0625 (the result of mating between first cousins or closer relatives) and 0.125 (the result of mating between half‐sibs or grandparent–grandchild or closer relatives), and 19 sheep had *F* values of 0.25 or greater, indicating that the closest form of inbreeding (i.e. between full‐sibs or parents and offspring) also occurs. Standard deviations were only moderately similar for the genomic estimates of inbreeding (*F*_hom_: 0.011, *F*_GRM_: 0.030, *F*_ROH_: 0.025), and 100% of individuals had positive nonzero inbreeding estimates for *F*_ROH_ (Fig. S1, Supporting information).

Of all the genomic measures, F_ROH_ showed the strongest correlation with *F*_ped_ for individuals with both parents and at least one maternal grandparent known (*r* = 0.71; Table 1, Fig. S2, Supporting information) and *F*_hom_ the weakest (*r* = 0.60; Table 1, Fig. S2, Supporting information), and the genomic measures all correlated more strongly with each other than with *F*_ped_. When the data set was restricted to individuals with four known grandparents, correlations between *F*_ped_ and the genomic measures increased substantially (up to *r* = 0.76), but were ranked in the same order as in the larger data set (Table 1). Genomic data allowed us to verify what proportion of individuals that are noninbred according to the pedigree are actually moderately closely inbred, defined as *F*_GRM_ or *F*_ROH_ higher than 0.1 We set this criterion in order to capture all individuals with *F*_ped_ of approximately 0.125 and higher, a commonly accepted criterion of moderately close inbreeding (Marshall *et al*. 2002) bearing in mind that *F*_GRM_ and *F*_ROH_ include variation around predicted *F*_ped_ and *F*_GRM_ is on a slightly different scale to *F*_ped_. Only 0.5% and 2.4% of noninbred individuals with at least one parent known were moderately or closely inbred according to the genomic estimators *F*_GRM_ or *F*_ROH_, and this decreased to 0.2% and 1.6%, respectively, for individuals with both parents and at least one maternal grandparent known, and to 0% and 1%, respectively, for individuals with all four grandparents known. This shows that cryptic moderate‐to‐close inbreeding only affects a minor proportion of the apparently noninbred individuals included in our analyses.

**Table 1:** Correlations between pedigree‐derived and genomic estimators of inbreeding

	*F*_ped_	*F*_GRM_	*F*_hom_	*F*_ROH_
*F*_ped_		0.72	0.67	0.77
*F*_GRM_	0.65		0.92	0.87
*F*_hom_	0.60	0.91		0.86
*F*_ROH_	0.71	0.86	0.83	

Values below the diagonal are Pearson's correlations for all individuals with at least both parents and one maternal grandparent known, and values above the diagonal show Pearson's correlations only including individuals with all four grandparents known.

### Inbreeding depression

**Figure 1:**
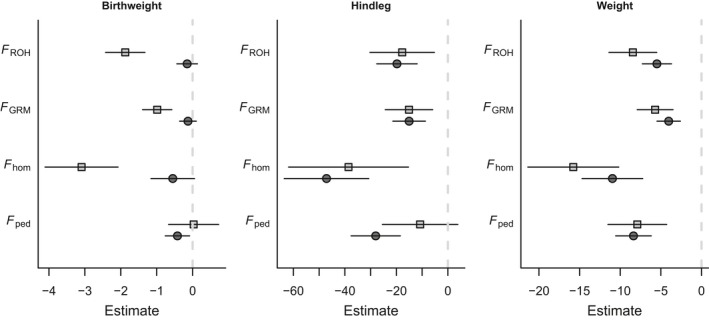
Estimates for pedigree and genomic inbreeding depression in juvenile body size. Light grey open squares indicate the slopes for maternal inbreeding depression, and dark grey closed circles indicate individual inbreeding depression. Error bars denote 1 standard error.

**Figure 2:**
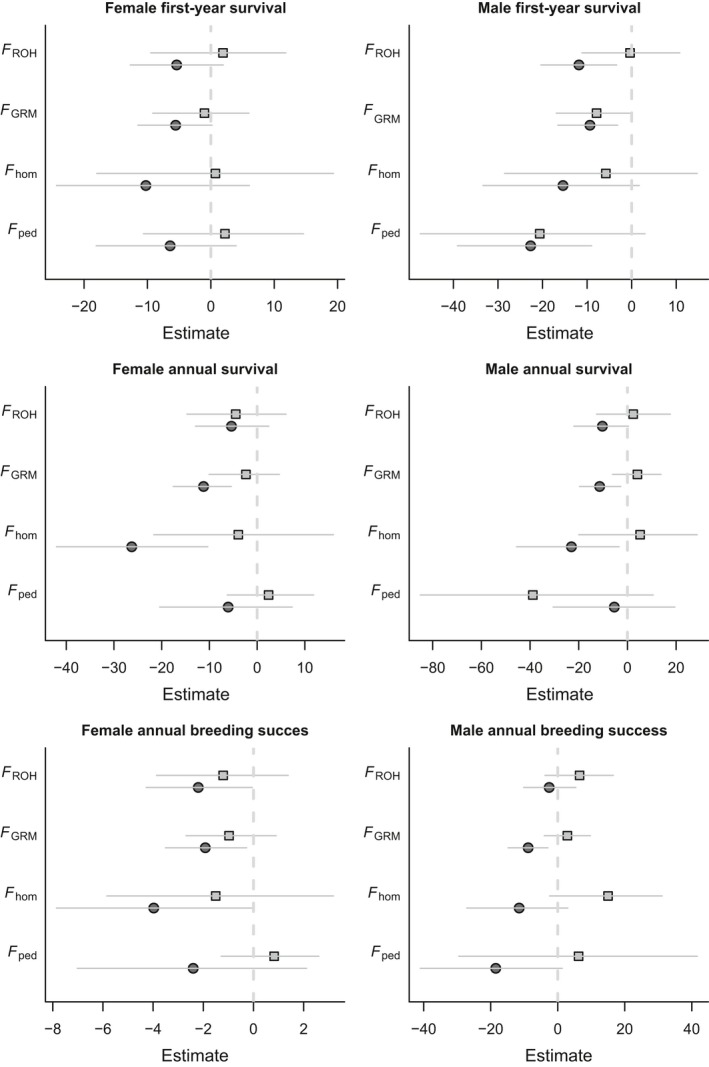
Estimates of (pedigree and genomic) inbreeding depression in sex‐specific first‐year survival, annual survival and annual breeding success. Light grey open squares indicate the slopes for maternal inbreeding depression, and dark grey closed circles indicate individual inbreeding depression. Error bars denote 95% credibility intervals.

Across all measure of inbreeding, we observed detrimental effects of being inbred in two of the three juvenile body size measures. An individual's own inbreeding coefficient was negatively correlated with hindleg and August weight, but not with birthweight (Fig. 1, Table 2). Inbreeding depression was also found when looking at fitness components, as individual inbreeding coefficient was negatively correlated with postyearling annual survival in both sexes, and with first‐year survival and (adult) annual breeding success in males (Fig. 2, Table 3).

**Table 2:** Parameter estimates from linear mixed models analysing the effects of individual and maternal inbreeding on lamb body size

Trait	Mean and standard deviation of trait value	Inbreeding coefficient	*N* (Pedigree)	*F*_ped_		*N* (Genomic)	*F*_hom_		*F*_GRM_		*F*_ROH_	
				Estimate	*P*		Estimate	*P*	Estimate	*P*	Estimate	*P*
Birthweight	2.08 (0.60)	Individual	2435	−0.42 (0.34)	0.212	2947	−0.55 (0.61)	0.362	−0.13 (0.23)	0.578	−0.15 (0.29)	0.595
		Maternal	650	0.03 (0.7)	0.967	727	−3.09 (1.02)	**0.002**	−0.99 (0.41)	**0.016**	−1.87 (0.55)	**0.001**
August hindleg	159.48 (10.49)	Individual	1449	−28.03 (9.55)	**0.003**	1706	−47.14 (16.41)	**0.004**	−15.05 (6.32)	**0.017**	−19.77 (7.84)	**0.012**
		Maternal	537	−10.78 (14.65)	0.462	591	−38.58 (23.28)	0.098	−15.1 (9.21)	0.102	−17.75 (12.52)	0.157
August weight	13.18 (2.76)	Individual	1454	−8.36 (2.18)	**<0.001**	1748	−10.96 (3.73)	**0.003**	−4.04 (1.44)	**0.005**	−5.48 (1.79)	**0.002**
		Maternal	543	−7.89 (3.61)	**0.03**	611	−15.77 (5.59)	**0.005**	−5.7 (2.21)	**0.01**	−8.44 (2.94)	**0.004**

Estimates are shown with the standard errors within parentheses. Birthweight and weight are measured in kilograms and hindleg in millimetres. Significant effects are shown in bold.

**Table 3:** Parameter estimates of GLMMs analysing the effects of individual and maternal inbreeding on annual fitness. Analyses were performed within‐sex

Trait	Inbreeding coefficient	*N* (Pedigree)	*F*_ped_		*N* (Genomic)	*F*_hom_		*F*_GRM_		*F*_ROH_	
			Estimate	*P*		Estimate	*P*	Estimate	*P*	Estimate	*P*
Female first‐year survival	Individual	1111	−6.42 (−18.08,3.99)	0.244	1430	−10.25 (−24.34,6.09)	0.188	−5.55 (−11.5,0.2)	0.06	−5.39 (−12.7,1.96)	0.154
	Maternal	483	2.23 (−10.63,14.62)	0.747	573	0.72 (−17.99,19.35)	0.967	−1.01 (−9.16,6.01)	0.826	1.92 (−9.48,11.83)	0.71
Female annual survival	Individual	508 (2448)	−5.58 (−19.37,6.56)	0.386	652 (3289)	−26.74 (−40.92, −10.86)	**<0.001**	−10.57 (−16.27, −4.48)	**<0.001**	−6.22 (−13.32,1.2)	0.098
	Maternal	307	2.37 (−6.28,12.21)	0.603	384	−1.34 (−20.7,14.85)	0.89	−1.47 (−9.22,5.36)	0.703	−3.79 (−13.3,6.47)	0.452
Female annual breeding success	Individual	508 (2448)	−2.31 (−6.94,2.28)	0.304	652 (3289)	−3.22 (−7.71,0.85)	0.131	−1.61 (−3.38,0.1)	0.067	−2.02 (−4.48,0.1)	0.078
	Maternal	307	0.87 (−0.98,2.83)	0.384	384	−2.09 (−6.57,2.39)	0.368	−1.12 (−3.05,0.6)	0.232	−1.3 (−3.89,1.17)	0.341
Male first‐year survival	Individual	996	−22.66 (−39.08, −8.99)	**<0.001**	1264	−15.41 (−33.36,1.7)	0.085	−9.36 (−16.58, −3.15)	**0.009**	−11.83 (−20.37, −3.38)	**0.002**
	Maternal	484	−20.63 (−47.43,3)	0.066	574	−5.8 (−28.5,14.7)	0.589	−7.87 (−16.91,0.02)	0.066	−0.37 (−11.14,10.78)	0.973
Male annual survival	Individual	370 (963)	−5.02 (−31.55,16.43)	0.679	466 (1259)	−23.11 (−45.43, −1.08)	**0.039**	−11.7 (−20.84, −2.87)	**0.009**	−12.21 (−24.27, −0.46)	**0.047**
	Maternal	256	−40.42 (−87.69,12.77)	0.104	314	5.89 (−17.97,32.45)	0.667	4.47 (−5.27,15.15)	0.378	2.62 (−13.39,17)	0.725
Male annual breeding success	Individual	370 (963)	−18.42 (−38.27,4.33)	0.087	466 (1259)	−10.73 (−26.54,4.68)	0.174	−8.22 (−13.98, −1.48)	**0.005**	−2.01 (−9.59,6.58)	0.651
	Maternal	256	6.25 (−29.3,40)	0.681	314	14.85 (−1.98,32.89)	0.099	2.64 (−4.18,9.84)	0.457	6.05 (−3.92,17.31)	0.259

95% Credible intervals for the parameter estimates are shown within parentheses. Sample sizes for each fitness measure are shown and give the total number of individuals, and the total number of observations for each fitness measure between parentheses. Significant effects are shown in bold.

Lambs born to inbred mothers were also significantly lighter both at birth and in August when they are approximately 4 months old (Fig. 2, Table 2) and had marginally nonsignificantly shorter hindlegs (Table 2). Maternal inbreeding coefficients had no significant effect on any of the annual fitness estimates, although its effect on male first‐year survival was marginally nonsignificant with *F*_ped_ and *F*_GRM_ (Fig. 2, Table 3).

When considering results obtained using the genomic estimators, the slopes of maternal inbreeding on juvenile weight were steeper than those for individual inbreeding (Fig. 1), indicating that for a lamb, having an inbred mother was worse than being inbred itself, while for hindleg estimates were more similar. Using the pedigree estimator, a different pattern was observed, as estimates for the maternal inbreeding slopes were typically shallower than for the individual slopes (Fig. 1), suggesting that the poorer pedigree information available for mothers hampers our ability to estimate maternal inbreeding depression. For the fitness measures, the estimates of slopes for the focal individual's inbreeding were universally steeper than those for their mother's inbreeding (Fig. 2), suggesting that by the time these traits are measured, being an inbred lamb is worse than having an inbred mother.

The data sets used in the *F*_ped_ and genomic analyses of inbreeding depression differed not only in sample size but also, given that this is a long‐term study, slightly in how the sheep were distributed in time, both of which might explain differences in results. Hence, we re‐analysed the effects of genomic inbreeding estimators on two juvenile body size traits, restricting the data set to individuals which were used in the *F*_ped_ analyses. Reducing the sample sizes led to increased standard errors around the estimates and higher *P* values, but point estimates for slopes changed very little compared to the larger data set (Table 2 and Table S4, Supporting information). *P* values obtained for genomic estimators were generally larger than for pedigree‐based estimates when studying individual inbreeding, but smaller when studying maternal inbreeding (Table S4, Supporting information). While genomic estimators were still able to detect inbreeding depression in the same traits as *F*_ped_ (Table S4, Supporting information), this analysis suggests that part of the increased detection of inbreeding depression in the main analyses (Tables 2 and 3) is due to the larger sample sizes available for the genomic estimators.

### Which inbreeding estimator estimates the most inbreeding depression

Having established that there is inbreeding depression in aspects of juvenile body size, first‐year survival and adult fitness, we were interested in how the various pedigree and genomic estimators of inbreeding compared in terms of the amount of inbreeding depression detected. Despite the moderate‐to‐strong correlations between them, there were striking differences between the various measures of inbreeding, and two patterns emerged. First, pedigree‐derived inbreeding detected fewer instances of a significant negative correlation between either maternal or individual inbreeding coefficients and body size or fitness than the genomic inbreeding estimates. As investigated above, this is partly but not wholly a consequence of slightly smaller samples size. Second, there was little difference between genomic estimators, for which sample sizes were identical for all analyses. Overall *F*_GRM_ showed the greatest ability to detect (significant) inbreeding depression in both body size and fitness as there was no trait for which inbreeding depression was detected using another estimator but not using *F*_GRM_. But interestingly, all genomic inbreeding estimates identified significant inbreeding depression for the exact same body size traits, and differences were only observed for the fitness measures. Differences in *P* values were very small, and there was a strong correspondence in the sign of the slope between estimators. We found that *F*_ROH_ estimates which were obtained using a longer threshold to define ROH (10 Mb) were highly correlated with *F*_ROH_ estimates presented here (*r* = 0.90, Fig. S3, Supporting information), but standard errors around the estimates of inbreeding depression widened considerably (Table S5, Supporting information).

## Discussion

We here provide evidence that inbreeding has severe consequences for juvenile body size, first‐winter survival and adult fitness in the Soay sheep. We show that being inbred has detrimental consequences for individuals themselves, as inbred sheep exhibit reduced juvenile body size, survival and number of offspring, but also for their offspring, as lambs born to inbred mothers are smaller than lambs born to noninbred mothers. In addition, we show that while genomic estimators of inbreeding are strongly correlated with pedigree‐derived inbreeding, genomic estimates of individual inbreeding have practical attributes making them superior to a typical wild pedigree in the detection of inbreeding depression.

### Inbreeding depression in Soay sheep

A previous study using a smaller sample size, a less complete pedigree and heterozygosity at up to 18 microsatellite loci failed to detect a clear pattern of inbreeding depression, either individual or maternal, in Soay sheep (Overall *et al*. 2005). One suggested explanation was that the mean and variance in inbreeding were very low in this population, which was in line with a commonly held belief that inbreeding is a rare phenomenon in vertebrate populations (Csilléry *et al*. 2006). We here document that over 40% of sheep with all four grandparents known have nonzero pedigree‐derived inbreeding coefficients, and even though moderate‐to‐close inbreeding is relatively rare (Fig. S1, Supporting information), this is clear evidence that mating between relatives is not uncommon in the study population. Despite this relatively common occurrence of inbreeding, inbreeding depression was only detected using pedigree *F* for three of nine traits, whereas genomic estimators of inbreeding enabled the detection of significant inbreeding depression in all bar two traits, suggesting that the importance of inbreeding depression in shaping variation in phenotype and fitness in the wild may often be underestimated by pedigrees with missing links. Additionally, all estimates of individual inbreeding depression were negative even if not significant in all cases (Tables 2 and 3), but taken together, this shows that inbreeding is probably affecting all traits included in this study.

Generally, it is believed that traits that are under stronger selection also exhibit stronger inbreeding depression (DeRose & Roff 1999). In Soay sheep, positive selection on weight is stronger than on leg length (Milner *et al*. 1999); hence, we would expect stronger inbreeding depression in the former. And indeed, when expressing the slopes in percentage change in trait value, we do observe this effect (Table 2). Inbreeding depression in weight (*F*_ped_ estimate: 6.3% change per 10% increase in inbreeding; *F*_GRM_ estimate: 3.1% change per 10% increase in inbreeding) was stronger than inbreeding depression in hindleg (*F*_ped_ estimate: 1.8% change per 10% increase in inbreeding; *F*_GRM_ estimate: 0.9% change per 10% increase in inbreeding) far stronger than average inbreeding depression in weight for livestock (Leroy 2014) and morphology in nondomestic animals (DeRose & Roff 1999).

Even stronger inbreeding depression would be expected for fitness components. Fitting the expectation, inbreeding depression was even stronger in first‐year survival, as after conditioning on differences in birthweight, a 10% increase in *F*_ped_ and *F*_GRM_ decreased first‐year survival by 65% and 63% in females and 90% and 72% in males, respectively (Table 3; note that this effect was only significant in males). Interestingly, when considering the genomic estimators, inbreeding depression was even stronger for adult (annual) survival than for first‐year survival (*F*_GRM_ estimates for females: −10.6 vs. −5.99, *F*_GRM_ estimates for males −12.53 vs. −9.62). This is at least a little surprising, given that inbreeding depression is often thought to be most severe in juvenile traits (DeRose & Roff 1999) and that inbred individuals suffer lower first‐year survival, although similar findings have been obtained in other systems (Keller *et al*. 2002). Perhaps this can be attributed to the fact that first‐year survival shows much stronger fluctuations between years than adult survival, as juveniles are more sensitive to differences in population density (Clutton‐Brock & Pemberton 2004). Hence, adult survival might be more directly influenced by individual quality and thus inbreeding status than first‐year survival.

Despite inbreeding depression in juvenile body size and survival rates, individuals are recruited to the breeding population that differ in inbreeding status. As, in mammals, maternal care is extensive and maternal effects on offspring quality are found in many systems (Rasanen & Kruuk 2007) including the Soay sheep (Jones *et al*. 2005; Wilson *et al*. 2005a), we investigated the consequences of variation in maternal inbreeding and found substantial effects. Lambs born to inbred mothers were smaller than lambs born to outbred mothers. Maternal inbreeding coefficients particularly affected birthweight and August weight, and the slopes were generally steeper than those of the regressions of an individual's own inbreeding coefficient on weight (Table 2). This implies that inbred mothers are not able to provision their offspring as well during gestation and/or lactation as more outbred mothers, presumably due to being in poorer condition. The observation that slopes for maternal inbreeding depression were steeper than those for individual inbreeding depression for the two weight measures could reflect stronger directional dominance for maternal inbreeding depression, suggesting stronger historical selection on maternal effects for juvenile weight than on the focal individual's own contributions to juvenile weight. Alternatively, selection may be operating more efficiently on genes carried by the focal individual, resulting in stronger purging of deleterious alleles (Glémin 2003).

Despite the negative effects of maternal inbreeding on juvenile weight, and the known negative association between juvenile body size and fitness (Milner *et al*. 1999; Wilson *et al*. 2005b), maternal inbreeding had only a marginally nonsignificant effect on male first‐year survival and no significant effect on female first‐year survival or adult annual fitness measures for either sex (Table 3). Re‐analysing the first‐year survival data with an interaction term between maternal inbreeding and sex revealed that indeed there was a tendency for maternal inbreeding effects to be stronger in males (*P* = 0.11). Our estimates of maternal inbreeding depression in first‐year survival can be considered conservative as they are independent of the effects of maternal inbreeding on birthweight, a trait which is strongly positively associated with first‐year survival (Jones *et al*. 2005). We can only speculate as to why a stronger effect of maternal inbreeding depression was observed for male offspring than for female offspring. However, it is in line with theories that predict that males are more costly to raise, thus requiring greater levels of parental investment (Clutton‐Brock *et al*. 1981), which potentially expose differences in condition between inbred and noninbred females.

Given that maternal effects are widely documented and have been observed in a wide range of taxa ranging from birds and animals to fish and even arthropods (Reznick *et al*. 1996; Beckerman *et al*. 2006; Rasanen & Kruuk 2007), and maternal inbreeding effects have been reported in laboratory settings and livestock (Carolino & Gama 2008), it is surprising that there is so little evidence for maternal inbreeding depression in the literature on wild vertebrates. There are studies which have described that maternal inbreeding has an effect on traits typically ascribed to the mother, such as brood survival, hatching rate or fledgling success (Szulkin *et al*. 2007; Laws *et al*. 2010) or hatching rate, but few papers have attempted to reveal the relative importance of both maternal and individual inbreeding on traits commonly regarded as offspring traits (body size, survival, breeding success).

A notable exception is a recent paper which described the effects of maternal and individual inbreeding on a range of life‐history and fitness traits in a population of red deer on the Isle of Rum (Huisman *et al*. 2016). For the traits which were analysed in both papers, some similarities emerge. For example, inbreeding depression was observed in first‐year or overwinter survival (red deer: both sexes combined, Soay sheep: only significant in males) and annual breeding success in males in both systems. There were also differences, as significant inbreeding depression in female annual breeding success and birthweight was only observed in red deer, and inbreeding depression in adult annual survival was only observed in Soay sheep. Maternal inbreeding was significantly associated with birthweight only in Soay sheep, whereas survival to recruitment age was affected by maternal inbreeding only in red deer. In part, these differences seem likely to reflect the different levels of investment by Soay and red deer mothers during gestation and lactation. Red deer have a substantially longer lactation than Soay sheep, and rearing an offspring, especially under higher densities, lowers the probability of producing a calf the following year in red deer but not in Soay sheep (Clutton‐Brock & Coulson 2002). The high cost of rearing offspring in red deer may make it more likely that inbreeding depression will be revealed in this trait in red deer than Soay sheep. However, more comparative information on sources of variation in fitness components, including inbreeding, across multiple species will be required to understand the patterns observed in different species in detail.

The lack of studies testing for maternal inbreeding depression is probably due to the fact that power to detect depression due to maternal inbreeding in a given data set is typically lower than for detecting depression due to inbreeding in the focal individuals. On the one hand, sample sizes are usually smaller for mothers than for focal individuals, and on the other hand, regardless of what restrictions are applied to ancestry information, pedigree depth is by definition one generation shallower for mothers. However, given that our results show that maternal inbreeding affects juvenile body size (a fitness‐related trait) at least as much as individual inbreeding, we would encourage workers, where possible, to test for the presence of such effects.

### Can genomic estimators of inbreeding be a substitute for pedigrees in the study of inbreeding depression?

Accurate estimates of pedigree inbreeding coefficients are difficult to obtain in natural populations (Pemberton 2008), and our results highlight that even in an intensively studied free‐living population with a relatively large amount of pedigree data and large sample sizes, pedigrees only capture a small proportion of the true variance in inbreeding, reducing the ability to detect inbreeding depression. The increased detection of inbreeding depression using genomic estimators is partly due to the fact that genomic estimates can be calculated for individuals with poor ancestry information. Another means through which genomic estimators of inbreeding are probably superior to pedigree inbreeding is through capturing the realized variance in inbreeding coefficients rather than the pedigree‐derived expected inbreeding coefficients. It is possible that even if recent ancestry is known perfectly for all individuals, genomic estimates may outperform pedigree‐derived estimates, as they are typically more strongly correlated with the proportion of the genome IBD (Kardos *et al*. 2015), although empirical studies which directly test this hypothesis are currently lacking for natural populations (Forstmeier *et al*. 2012). Our results do not support this, however, as when we restricted our data set for genomic estimators to individuals included in the pedigree analyses, slopes became shallower and standard errors increased, resulting in no net statistical gain over the analyses performed using the pedigree (Table S2, Supporting information).

Supporting a previous study, our results confirm that detection of inbreeding depression using molecular data alone is now completely justified (Hoffman *et al*. 2014; Huisman *et al*. 2016) as genomic estimators are at least as capable of detecting inbreeding depression as pedigrees. The degree to which genomic estimators prove to be better predictors than pedigree inbreeding probably depends on pedigree quality, pedigree size, the number and distribution of recombination events and potentially the frequency of mating between (close) relatives, meaning that in systems where pedigrees are deep and near‐perfect (Sardell *et al*. 2010) and inbreeding is relatively common, genomic estimators might not have a substantial advantage over pedigree inbreeding. However, availability of genomic estimators should not replace attempts at genetic inference of parentage, as highlighted by the importance of inbreeding depression due to maternal inbreeding and inbreeding depression in annual breeding success in both sexes.

Genomic estimators inherently differ in scale both from each other and from pedigree inbreeding. This poses us with a problem when we are interested in the effects of inbreeding [e.g. the result of full‐sib mating (*F *=* *0.25)] on fitness compared to fitness shown by outbred individuals (*F *=* *0). However, except for *F*_hom_, the genomic estimators here are fairly comparable to *F*_ped_. For the most common pedigree inbreeding classes, mean *F*_ROH_ and *F*_GRM_ are relatively similar to the *F*_ped_ estimates, even though there is substantial variance around the mean (Fig. S2, Supporting information). For many purposes, however, the absolute values of *F* (or regressions of fitness on *F*) are not of great importance and a more pragmatic approach suffices, for example if we are interested in whether fitness‐related traits decrease significantly with estimates of *F* (Wang 2014).

### Which genomic method performs best?

In the literature, there is little consensus on which genomic estimator of inbreeding scientists studying inbreeding depression in the wild should use. Simulations showed that in humans *F*_ROH_ should correlate more strongly with the mutational load than any other genomic estimate (Keller *et al*. 2011), but simulations using a broader set of population parameters showed that other genomic estimates were equally good or better estimators for the true proportion of the genome IBD than *F*_ROH_ under a range of conditions (Kardos *et al*. 2015). We here demonstrate that while *F*_GRM_ detected inbreeding depression the most consistently across both juvenile body size traits and fitness, there is very little difference in performance between the genomic estimators. In the field of human genetics, ROH has gained considerable traction and is the most commonly, and often the only, estimator used to test for the existence of inbreeding depression (McQuillan *et al*. 2012; Verweij *et al*. 2014; Joshi *et al*. 2015). However, intriguingly, in a paper which explored the effects of both homozygosity and *F*_ROH_ on human stature, *F*_hom_ performed equally well, and even after controlling for *F*_ROH_, had a significant effect (McQuillan *et al*. 2012). In dairy cattle, with an effective population size more similar to the Soay sheep than that of humans, the use of *F*_GRM_ resulted in more cases of significant inbreeding depression than *F*_ROH_, a pattern which emerged for both production and fitness traits (Bjelland *et al*. 2013).

Our results show that if marker density is sufficiently high, most genomic estimators perform at least as well as pedigree‐derived inbreeding coefficients, especially if pedigrees are incomplete. But there are inevitably subtle differences in how various estimators correlate with inbreeding and autozygosity at loci harbouring (partially) recessive deleterious alleles, and thus in how powerful they are as estimators in the detection of inbreeding depression. Nonetheless, establishing which genomic estimator to use in the analysis of inbreeding depression is not an easy task, but can prove a crucial factor as to whether significant inbreeding depression is or is not detected. Based on simulation studies (Keller *et al*. 2011; Kardos *et al*. 2015), it is probably that the relative performance of the various genomic correlates depends on demography (past and current effective population size, mating structure, spatial population structure) and marker density relative to linkage disequilibrium. In our study, *F*_hom_ and *F*_GRM_ are strongly correlated between nonoverlapping sets of markers and asymptote at about half the total number of marker, indicating that adding more markers would not improve our genomic estimates of inbreeding coefficients much (Fig. S4, Supporting information). This result is supported by the presence of significant Identity Disequilibrium measured by the estimator *g*_2_ in the r package *InbreedR* (David *et al*. 2007; Stoffel *et al*. 2015). The estimator *g*_2_ was estimated at 0.0014 ± 0.0002 SD (*P* = 0.001, Fig. S6, Supporting information), which is exceedingly low compared to a meta‐analyses which obtained *g*_2_ estimates from 50 published HFC studies (Miller & Coltman 2014) and to estimates obtained using RAD‐seq data in stranded harbour seals (Hoffman *et al*. 2014) but very similar to estimates from the long‐term study of the red deer on the Isle of Rum (Huisman *et al*. 2016). We have also examined the effects of marker number on the estimates of maternal and individual inbreeding depression for August weight and found that using 30% of the markers was sufficient to reliably estimate inbreeding load using *F*_hom_ and *F*_GRM_ (Fig. S5, Supporting information). The Soay sheep population, being a primitive remnant island population, has small N_e_ (Kijas *et al*. 2012), high LD, and mating between relatives is relatively common. Detection of inbreeding depression in large panmictic populations may well require substantially larger marker panels and the preferred genomic estimator may be different from the one preferred here (*F*_GRM_). However, inbreeding depression is of great concern for many endangered organisms and other species with small population sizes (Hedrick & Kalinowski 2000), and in line with Hoffman *et al*. (2014), our results suggest that marker numbers which are easily achievable either using RAD‐Seq or a SNP chip could realistically be used to detect inbreeding depression in such populations alongside or instead of a pedigree. More empirical work exploring how these various conditions affect the ability of genomic estimators to detect inbreeding depression is clearly needed. In the meantime, it may be advisable for researchers to, where possible, examine the performance of several genomic estimators instead of only relying on one.

In conclusion, we have demonstrated that maternal inbreeding has an effect on both juvenile body size and first‐year survival in a free‐living population of Soay sheep and that inbreeding depression is found in juvenile body size and both juvenile and adult fitness. We examined the performance of several genomic inbreeding estimators, and they were all found to detect more inbreeding depression than pedigree‐derived inbreeding coefficients. This result was partly due an increase in available sample size as genomic estimators could be calculated for individuals with poor pedigree information. Our results suggest that moderate‐to‐high density marker information can successfully be used to estimate inbreeding depression in populations for which reconstructing multigenerational pedigrees is impractical.

J.G.P and J.M.P. organized the long‐term collection of phenotypic data and DNA samples, C.B. and P.A.E. performed laboratory work, C.B analysed the data, C.B and J.M.P wrote the manuscript, all authors have approved the final version..

## Data accessibility

Inverse pedigree relatedness matrix, pedigree and genomic inbreeding coefficients, fitness data, body size data, covariates and categorical variables included in the models are archived on Dryad (doi: 10.5061/dryad.fh6d9).

## Supporting information


**Appendix S1.** Restrictions on ancestry information when calculating pedigree‐derived inbreeding.
**Table S1.** The effects of imposing different levels of minimum ancestry information on total sample size and the total number of inbred individuals.
**Table S2.** Parameter estimates from linear mixed models analysing inbreeding depression in juvenile body size.
**Fig. S1.** Histograms showing the distributions for pedigree‐derived inbreeding and the three genomic estimators of inbreeding used.
**Fig. S2.** Pairwise correlations between pedigree‐derived inbreeding and three genomic estimators of inbreeding.
**Fig. S3.** Pairwise correlations between pedigree‐derived inbreeding and genomic estimators of inbreeding.
**Table S3.** Fixed and random effects fitted in the models analysing inbreeding depression in juvenile body size and fitness.
**Table S4.** Parameter estimates from linear mixed models analysing the effects of inbreeding on August body size in lambs.
**Table S5.** Parameter estimates from linear mixed models analysing the effects of the proportion of the genome in runs of homozygosity (ROH) on August lamb body size.
**Appendix S2.** Correlations in heterozygosity among loci and idenity disequilibrium.
**Fig. S4.** The effect of number of SNPs on (a) the correlation between pedigree *F* and SNP‐by‐SNP based inbreeding estimators (*F*_hom_ and *F*_GRM_), and (b) the correlation in SNP based inbreeding estimators in one half of the markers with SNP based inbreeding estimators in the other half.
**Fig. S5.** The effect of SNP marker number on the correlation of offspring and maternal genomic inbreeding estimators (*F*_hom_ and *F*_GRM_) with August weight in lambs.
