# Supplementary material for: Genomic analysis reveals depression due to both individual and maternal inbreeding in a free‐living mammal population
Source: Mol Ecol. 2016 Jun 6;25(13):3152–68. doi: 10.1111/mec.13681 (PMC4950049; doi:10.1111/mec.13681)
Supplement: Supplementary file 1 — Appendix S1. Restrictions on ancestry information when calculating pedigree‐derived inbreeding. Table S1. The effects of imposing different levels of minimum ancestry information on total sample size and the total number of inbred individuals. Table S2. Parameter estimates from linear mixed models analysing inbreeding depression in juvenile body size. Fig. S1. Histograms showing the distributions for pedigree‐derived inbreeding and the three genomic estimators of inbreeding used. Fig. S2. Pairwise correlations between pedigree‐derived inbreeding and three genomic estimators of inbreeding. Fig. S3. Pairwise correlations between pedigree‐derived inbreeding and genomic estimators of inbreeding. Table S3. Fixed and random effects fitted in the models analysing inbreeding depression in juvenile body size and fitness. Table S4. Parameter estimates from linear mixed models analysing the effects of inbreeding on August body size in lambs. Table S5. Parameter estimates from linear mixed models analysing the effects of the proportion of the genome in runs of homozygosity (ROH) on August lamb body size. Appendix S2. Correlations in heterozygosity among loci and idenity disequilibrium. Fig. S4. The effect of number of SNPs on (a) the correlation between pedigree F and SNP‐by‐SNP based inbreeding estimators (F hom and F GRM), and (b) the correlation in SNP based inbreeding estimators in one half of the markers with SNP based inbreeding estimators in the other half. Fig. S5. The effect of SNP marker number on the correlation of offspring and maternal genomic inbreeding estimators (F hom and F GRM) with August weight in lambs. [file MEC-25-3152-s001.pdf]

Table of Contents

Restrictions on ancestry information when calculating pedigree-derived inbreeding...............2

Supplementary Figures 7

Supplementary Tables..10

Correlations in heterozygosity among loci 13

Restrictions on ancestry information when calculating pedigree-derived inbreeding

Introduction

The Soay sheep pedigree, like virtually all pedigrees constructed for free-living populations, contains gaps. Only ca. 30% of the island population is intensively studied, meaning that there are immigrants to the study area and that not all candidate parents are sampled. Of the 6,336 non-founder individuals in the pedigree, there are 4238 individuals for which both parents are known, and this number decreases further when restricting the dataset to only include individuals with all four grandparents known (n=1,591). When using pedigree *F*, incomplete ancestry information means that inbred individuals are falsely considered noninbred. Hence it might intuitively make sense to restrict analyses to only include individuals that have a lot of ancestry information or more complete ancestry information than a specific threshold. As shown above, such a restriction will come at a cost, as sample size will decline. Additionally, imposing increasingly stringent criteria could also reduce the sample size of inbred individuals, if they do not meet the restriction criteria.

Methods

We examined what the most appropriate data restriction criteria would be for our dataset by comparing the occurrence of inbreeding in the following four datasets: 1) all individuals with at least one parent known, 2) all individuals with both parents known; 3) all individuals with both parents and at least one maternal grandparent known, 4) all individuals with both parents and both maternal grandparents known, and 5) all individuals with all four grandparents known. Note that since female Soay sheep rarely disperse from their natal area, informaton on maternal grandparents is generally more complete than for paternal grandparents.

As a consequence of the above (results presented below), for our main analyses we restricted the dataset to only include individuals with both parents known and at least one maternal grandparent known. Restrictions were only imposed on the offspring, such that the consequences of maternal inbreeding were estimated in a subset of mothers that had at least one parent known. As using such relaxed restrictions could underestimate close inbreeding events in individuals and, to an even larger extent in mothers, this could potentially affect estimates of the maternal inbreeding effects. Hence, we also re-analysed the three early-life morphological traits in subsets of the data created using two additional criteria: in a dataset only containing 1) focal individuals and mothers with two parents known, and 2) focal individuals with all four grandparents known.

Results

As expected, the size of the total dataset decreased with increasingly stringent criteria (Table S1). And while inbred individuals made up a larger proportion when more stringent criteria were applied, the total number of inbred individuals decreased too, the effect of which was especially pronounced when all four grandparents are required (Table S1). Interestingly, a reduction in sample size was seen not only for relatively highly inbred individuals, but also particularly for mildly inbred individuals ($F<0.0625$). From this, it emerges that the probability of falsely calling an inbred individual non-inbred needs to be weighed against the total sample size of inbred individuals. Even when all four grandparents are known 5.7% of individuals have inbreeding coefficients of 0.0625 or greater. Therefore, even if 5.7% of closely inbred individuals were wrongly classified as non-inbred when using less stringent criteria, it is unlikely that this downwards bias in inbreeding coefficients would have a large

effect on inbreeding depression estimates. In order to obtain a dataset with as many individuals with non-zero inbreeding coefficients as possible while simultaneously ensuring sufficient ancestry information, for our main inbreeding depression analyses we chose to include individuals with both parents and at least the maternal grandmother known.

Estimating inbreeding depression with incomplete pedigrees, resulting in underestimated individual F values is problematic, as a sizeable proportion of individuals with F=0 are probably inbred to some degree. We therefore re-estimated the effect of F on juvenile body size using 1) offspring and mothers with both parents known ($F_{ped,both}$), and 2) using offspring with all 4 grandparents known ($F_{ped,4grandparents}$). The smaller but more accurate dataset of $F_{ped,both}$ detected significant individual and maternal inbreeding depression in every single case it was detected by F_{ped}, but P values were higher (Table S2). Using the even more complete and smaller dataset of $F_{ped,4grandparents}$, no significant effect could be detected in any of the traits (Table S2). In general, as ancestry criteria became more stringent, the estimated slopes of trait values on inbreeding coefficients became shallower and standard errors larger (Table S2).

Table S1: The effects of imposing different levels of minimum ancestry information on total sample size and the total number of inbred individuals.

Dataset restriction	Total number of individuals	Total number of inbred (*F*>0) and noninbred individuals	Individuals with *F* >0 and < 0.0625	Individuals with *F* >=0.0625 and < 0.125	Individuals with *F* >=0.125 and < 0.25	Individuals with *F* >=0.25	Mean *F*	Standard deviation in *F*
At least one parent known	6336	813 / 5523	701	58	35	19	0.003	0.018
Both parents known	4238	813 / 3425	701	58	35	19	0.005	0.022
Both parents and at least one maternal grandparent known	3816	811 / 3005	701	58	34	18	0.005	0.023
Both parents and both maternal grandparents known	3040	744 / 2296	637	57	34	16	0.006	0.024
All four grandparents known	1591	656 / 935	566	51	32	8	0.009	0.028

Table S2: Parameter estimates from linear mixed models analysing inbreeding depression in juvenile body size. Estimates are shown for models using individuals with two parents known and a mother with at least one parent known (F_{ped}), using individuals and mothers with two parents known each ($F_{ped,both}$) and models only using individuals with at least four grandparents known ($F_{ped,4grandparents}$). Estimates include standard errors within parentheses.

Trait	Inbreeding	Mean and standard deviation of trait value	F_{ped}			$F_{ped,both}$			$F_{ped,4grandparents}$		
			Estimate	P	N	Estimate	P	N	Estimate	P	N
Birthweight	Individual	2.08 (0.60)	-0.42 (0.34)	0.212	2435	-0.39 (0.33)	0.245	1911	-0.43 (0.41)	0.29	948
	Maternal		0.03 (0.7)	0.967	650	0.03 (0.72)	0.963	504	-0.66 (0.97)	0.496	388
Hindleg	Individual	159.48 (10.49)	-28.03 (9.55)	**0.003**	1449	-25.7 (9.9)	**0.01**	1159	-15.09 (12.08)	0.212	586
	Maternal		-10.78 (14.65)	0.462	537	-10.2 (15.23)	0.503	430	-3.7 (25.35)	0.884	311
Weight	Individual	13.18 (2.76)	-8.36 (2.18)	**<0.001**	1454	-7.49 (2.2)	**0.001**	1157	-5.25 (2.68)	0.051	585
	Maternal		-7.89 (3.61)	**0.03**	543	-7.46 (3.64)	**0.041**	430	-6.71 (5.76)	0.245	311

Supplementary Figures

Fig S1: Histograms showing the distributions for pedigree-derived inbreeding and the three genomic estimators of inbreeding used.

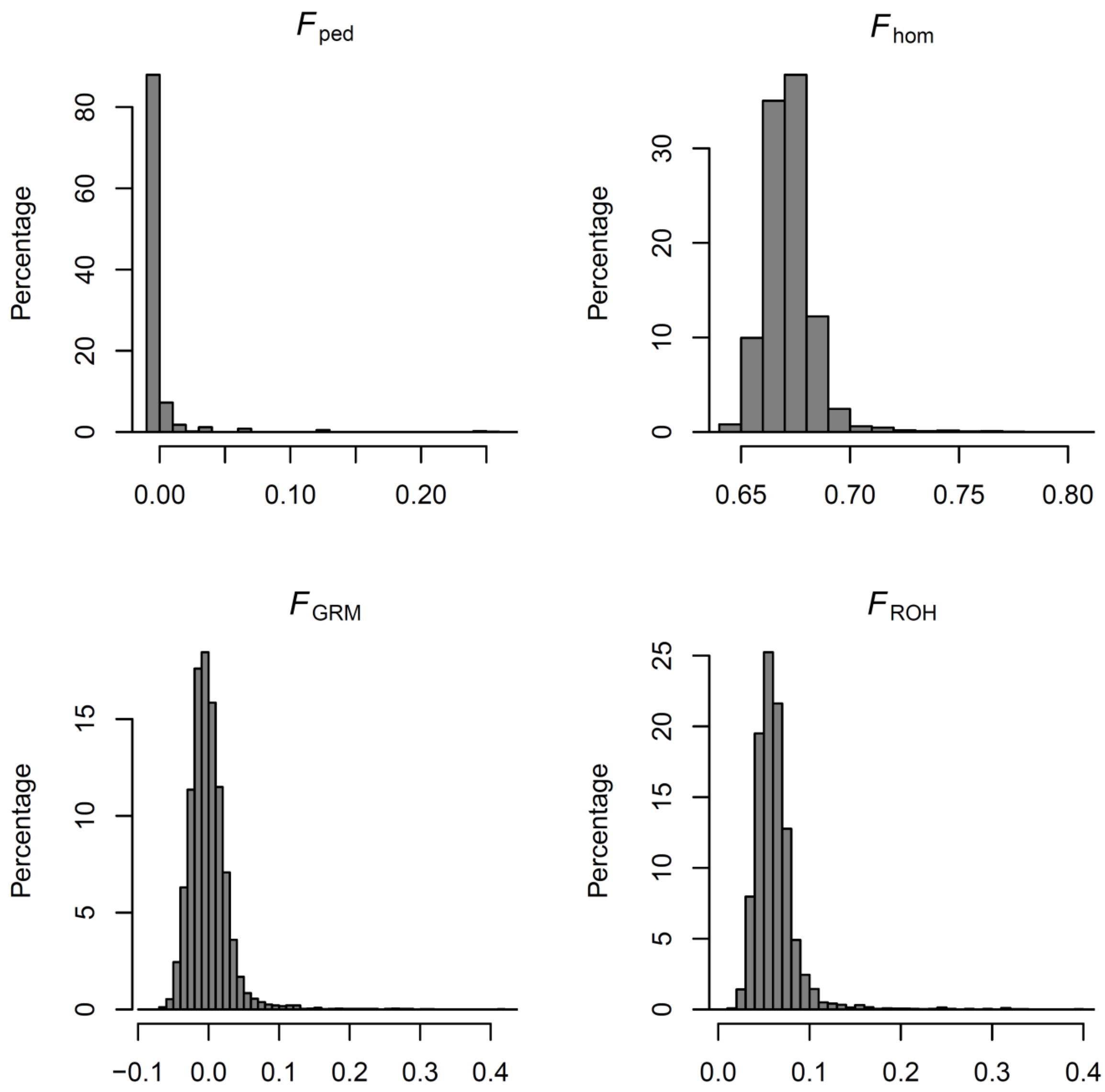

Fig S2: Pairwise correlations between pedigree-derived inbreeding and three genomic estimators of inbreeding. Values above the diagonal show the Pearson's correlations and associated P values. The dataset was restricted to individuals with both parents and at least one maternal grandparent known.

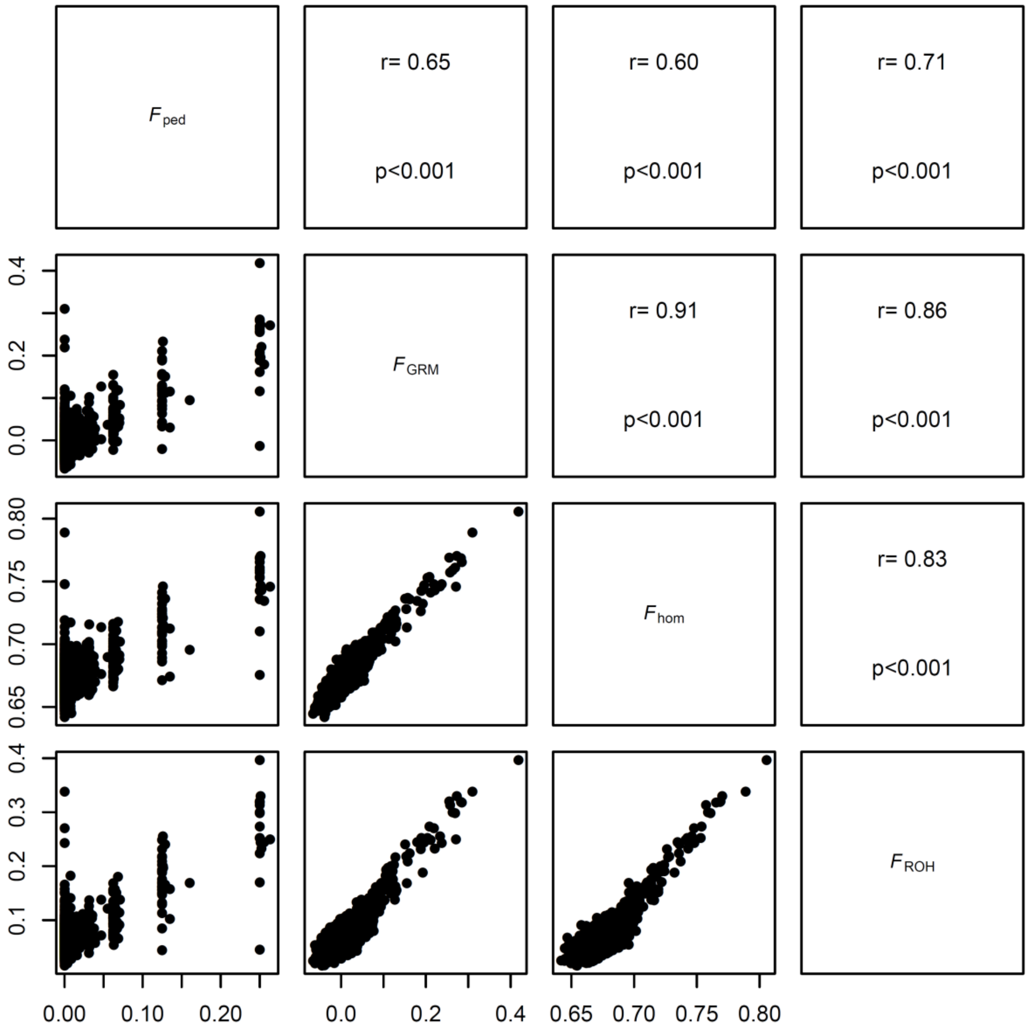

Fig. S3: Pairwise correlations between pedigree-derived inbreeding and genomic estimators of inbreeding. Criteria for calling runs-of-homozygosity (ROH) are arbitrary, and different criteria may lead to different results. We thus explored whether setting a longer minimum detection threshold of 10Mb would make a difference in our results. When using this criterion ($F_{\mathrm{ROH,10Mb}}$) the proportion of individuals with non-zero F decreased from 100% for F_{roh} to 93.1% for $\mathrm{F_{roh10K}}$, while the standard deviation decreased from 0.025 to 0.020. $F_{\mathrm{ROH,10Mb}}$ correlated strongly with F_{ROH} (r=0.91), and was more strongly correlated with F_{ped} and more weakly correlated with the other SNP by SNP-based estimates. Values above the diagonal show the Pearson's correlations and associated P values. The dataset was restricted to individuals with both parents and at least one maternal grandparent known.

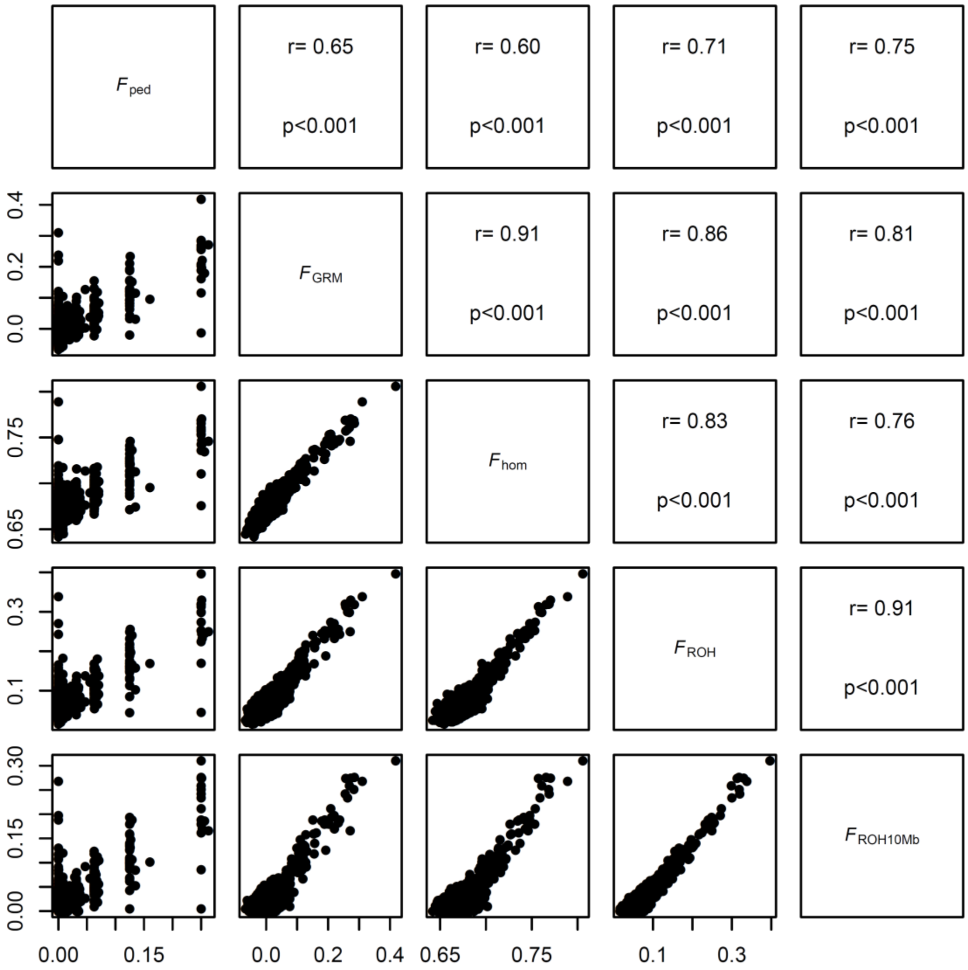

Supplementary Tables

Table S3: Fixed and random effects fitted in the models analysing inbreeding depression in juvenile body size and fitness. Tick marks denote whether a specific term was included in the model.

	Fixed effects						Random effects					
**Trait**	**Sex (Male or female)**	**Litter Size (0 or 1)**	**Maternal age (years, quadratic)**	**Age (years, quadratic)**	**Age at capture (Factor)**	**Adjusted birth weight (Kg)**	**Year of Birth**	**Sheep Year**	**Sheep ID**	**Maternal ID**	**Additive genetic effect**	**Maternal genetic effect**
**Birth weight**	x	x	x		days		x				x	x
**Hindleg**	x	x	x		months		x				x	x
**Weight**	x	x	x		months		x				x	x
**First year survival**		x	x	x		x	x			x		
**Annual survival**				x			x	x		x		
**Annual breeding success**				x			x	x	x	x		

Table S4: Parameter estimates from linear mixed models analysing the effects of inbreeding on August body size in lambs. For the genomic measures (F_{GRM} , F_{ROH} and F_{hom}) only individuals included in the F_{ped} analyses were used. Estimates are shown with standard errors within parentheses.

Trait	Inbreeding	F_{ped}		F_{GRM}		F_{ROH}		F_{hom}	
		Estimate	P	Estimate	P	Estimate	P	Estimate	P
Hindleg	Individual	-28.03 (9.55)	**0.003**	-13.22 (6.89)	0.055	-18.28 (8.44)	**0.031**	-41.19 (17.99)	**0.022**
	Maternal	-10.78 (14.65)	0.462	-14.7 (10.07)	0.145	-18.06 (13.54)	0.183	-46.74 (25.3)	0.065
Weight	Individual	-8.36 (2.18)	**<0.001**	-3.74 (1.56)	**0.017**	-5.47 (1.91)	**0.004**	-9.07 (4.08)	**0.026**
	Maternal	-7.89 (3.61)	**0.03**	-6.7 (2.41)	**0.006**	-9.12 (3.18)	**0.004**	-19.04 (6.08)	**0.002**

Table S5: Parameter estimates from linear mixed models analysing the effects of the proportion of the genome in runs of homozygosity (ROH) on August lamb body size. We here compared the regressions of $F_{ROH,10Mb}$ and F_{ROH} on the three August body size traits, using the exact same model structure. P values of $F_{ROH,10Mb}$ were generally higher than those of F_{ROH} confirming that using a minimum threshold of 5Mb captures (partially) recessive deleterious alleles better than using a longer threshold. Estimates from models using ROH > 5Mb (F_{ROH}) or ROH > 10Mb ($F_{ROH,10Mb}$) are shown with standard errors within parentheses.

		F_{ROH}		$F_{ROH,10Mb}$	
**Trait**	**Inbreeding**	**Estimate**	**P**	**Estimate**	**P**
Hindleg	Individual	-19.77 (7.84)	**0.012**	-23.74 (9.73)	**0.015**
	Maternal	-17.75 (12.52)	0.157	-21.77 (16.65)	0.192
Weight	Individual	-5.48 (1.79)	**0.002**	-5.1 (2.23)	**0.022**
	Maternal	-8.44 (2.94)	**0.004**	-6.92 (3.85)	0.073

Correlations in heterozygosity among loci and Idenity Disequilibrium

Heterozygosity-heterozygosity correlations

The strength of the correlation between genomic estimators of inbreeding and *F*, and thus its power to detect inbreeding depression is dependent on the number of markers used (Balloux *et al.* 2004; Slate *et al.* 2004). Consequently, if genomic inbreeding estimators and *F* are correlated, within-individual genomic inbreeding coefficients at marker loci should be correlated (Balloux *et al.* 2004). We tested for the strength of the correlation in F_{hom} and F_{GRM} by randomly dividing the genetic data into two non-overlapping sets of SNP markers, and testing whether within-individual F_{hom} and F_{GRM} with one set of markers correlated with within-individual F_{hom} and F_{GRM} measured with the other set of loci. This process was repeated 50 times. We then assessed 1) whether the strength of the correlation increased with the number of markers used, and 2) if the strength of the correlation saturated at or before the currently available number of markers. If the strength of the correlation asymptoted with increasing marker number, this would indicate that we had captured most of the variation in inbreeding with the available markers. To test this, we randomly sampled 1%, 5%, 10%, 30%, 50%, 70% and 90% of the available markers, repeated this 50 times, and calculated both F_{hom}-F_{hom} and F_{GRM}-F_{GRM} correlations for each set of markers.

The correlation between SNP-by-SNP based inbreeding estimators (F_{hom} and F_{GRM}) and pedigree *F* increases with the number of markers used (Fig S4a), but the improvement is only marginal once 50% of the markers are used. When splitting the markers into two equal subsets, F_{hom} and F_{GRM} in one half of the markers correlates with F_{hom} and F_{GRM} in the other half, and the strength of this correlation increases with increasing number of total markers (Fig S4b), increasing to 0.92 and 0.94 when all the markers are used for F_{hom} and F_{GRM}

respectively. Similar to the correlation with pedigree *F*, adding more markers has a larger effect on the correlations between marker sets when marker number is low than when marker number is high. In both analyses, when the same number of SNP markers is used, the correlations for F_{GRM} are stronger than those for F_{hom} (Fig. S3).

Fig.S4: The effect of number of SNPs on a) the correlation between pedigree *F* and SNP-by-SNP based inbreeding estimators (F_{hom} and F_{GRM}), and b) the correlation in SNP based inbreeding estimators in one half of the markers with SNP based inbreeding estimators in the other half. Box and whiskers indicate the median and spread observed for 50 replicate models where markers were sampled at random. Correlations for F_{hom} and F_{GRM} are shown in dark grey and light grey respectively.

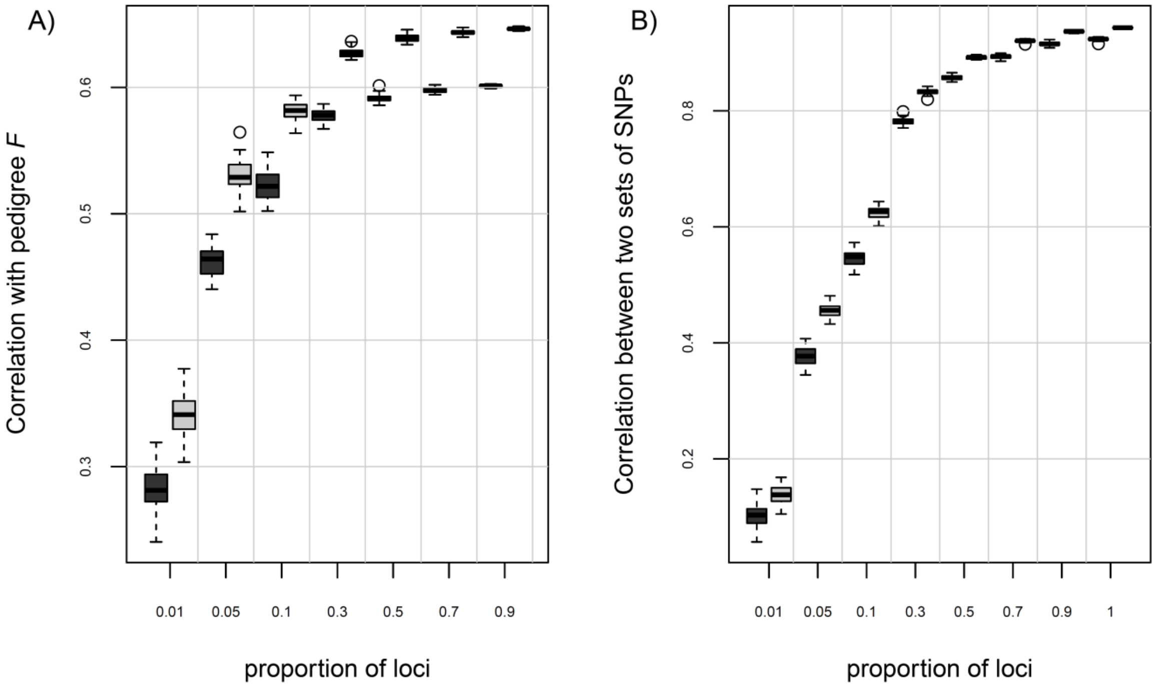

Identity Disequilibrium

While heterozygosity-heterozygosity correlations are a valuable exercise as tghey can show us how strongly correlated heterozygosity estimates are between subsets of markers, the tests aren't fully independent which makes testing of significance problematic. A better approach is to test for Identity Disequilibrium (ID) between loci using the g_2 estimator (David *et al.* 2007; Szulkin *et al.* 2010) which estimates the covariance in heterozygosity between markers standardised by mean heterozygosity and which does not depend on the loci used. Hence we estimated g_2 in the R package *InbreedR* (Stoffel *et al.* 2015). Confidence intervals were obtained by bootstrapping (n=100) and significance was assessed using permutations (n=100). We estimated g_2 for individuals with both parents and one maternal grandparent known, but due to memory constraints we restricted our analysis to a subset of 1500 randomly sampled individuals. In the same package, we also estimated the expected correlation between heterozygosity and inbreeding, and bootstrapping was perfomed 100 times.

We show that while there is significant ID, g_2 is very low (0.0014 +/ 0.0002 SD, P =0.01). Our estimate for the Soay sheep is substantially lower than estimates obtained using RAD-seq data in harbour seals (Hoffman *et al.* 2014) and much lower than estimates in a meta-analysis which estimated g_2 in 50 published HFC studies (Miller & Coltman 2014). Interestingly, the estimate for the Soay sheep is very similar to estimates obtained using a similar number of SNPS for the population of red deer on the Isle of Rum (Huisman *et al.* (in press)). Our low estimates are not a result of insufficient marker density, as we show that the expected correlation between heterozygosity and inbreeding is very high (r2= 0.963, 95% CI: 0.955, 0.974). Instead, low g_2 can likely be explained by the relatively rare occurrence of close inbreeding in this population (Fig. S1).

The effect of varying marker number on inbreeding depression estimates

We then examined if and how estimates of inbreeding depression depended on the number of markers used to calculate F_{hom} and F_{GRM}. We again sampled 1%, 5%, 10%, 30%, 50%, 70% and 90% of the available markers, and with each set of markers we analysed the effects of individual and maternal F_{hom} or F_{GRM} coefficients on August weight in lambs using the same model structure as described in the main paper. This procedure was performed 50 times. We have used August weight as a proof of principle, as this trait showed evidence for both maternal and individual inbreeding depression.

The slopes for both maternal and individual inbreeding depression in lamb August weight steepened with increasing marker number (Fig. S5). Similarly, the absolute Z ratio (the ratio of slope: standard error) increased as more markers were used. Although mean Z ratios did not change a lot once 50% of the markers were used, the effects of sampling variance decreased substantially with increasing marker number (as shown by the decreasing spread of the box-and whisker plots in Fig. S5). Due to the large sampling error at lower marker numbers, some significant effects are detected even when using 1% if the markers (370 SNPs), but it is only when using 30% of the markers (11111 SNPs) that significant effects are picked up regardless of which SNPs are used in the genomic inbreeding estimators (Fig. S5).

Fig. S5: The effect of SNP marker number on the correlation of offspring and maternal genomic inbreeding estimators (F_{hom} and F_{GRM}) with August weight in lambs. Box and whiskers indicate the median and spread observed for 50 replicate models where markers were sampled at random. Increasing the number of SNPs leads to a steepening slope of the regression of weight on genomic inbreeding estimators (top row) and larger absolute Z ratios (bottom row). Values below the dashed lines (at a Z ratio of -2) in the bottom row reflect a highly likely significant effect of inbreeding. Results for F_{hom} and F_{GRM} are shown in dark grey and light grey respectively.

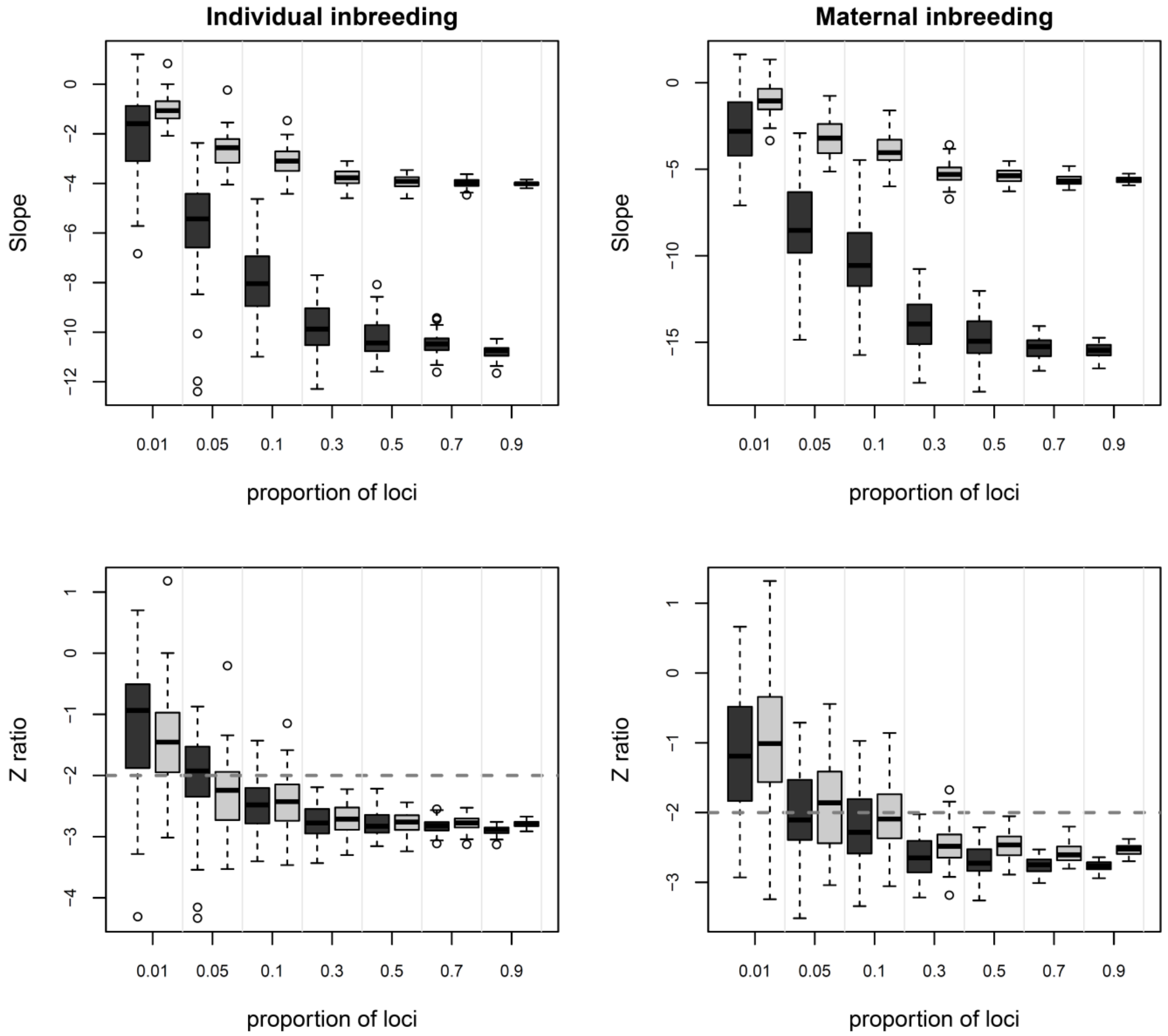

References

Balloux F, Amos W, Coulson T (2004) Does heterozygosity estimate inbreeding in real populations? *Molecular Ecology* **13**, 3021-3031.

David P, Pujol B, Viard F, Castella V, Goudet J (2007) Reliable selfing rate estimates from imperfect population genetic data. *Molecular Ecology* **16**, 2474-2487.

Hoffman JI, Simpson F, David P*, et al.* (2014) High-throughput sequencing reveals inbreeding depression in a natural population. *Proceedings of the National Academy of Sciences* **111**, 3775-3780.

Huisman J, Kruuk LEB, Ellis PA, Clutton-Brock T, Pemberton JM ((in press)) Inbreeding depression across the lifespan in a wild mammal population.

Miller JM, Coltman DW (2014) Assessment of identity disequilibrium and its relation to empirical heterozygosity fitness correlations: a meta-analysis. *Molecular Ecology* **23**, 1899-1909.

Slate J, David P, Dodds KG*, et al.* (2004) Understanding the relationship between the inbreeding coefficient and multilocus heterozygosity: theoretical expectations and empirical data. *Heredity* **93**, 255-265.

Stoffel MA, Esser M, Nichols H*, et al.* (2015) inbreedR: An R package for the analysis of inbreeding based on genetic markers},.

Szulkin M, Bierne N, David P (2010) Heterozygosity-fitness correlations: a time for reappraisal. *Evolution* **64**, 1202-1217.
